# Tumour vasculature immaturity, oxidative damage and systemic inflammation stratify survival of colorectal cancer patients on bevacizumab treatment

**DOI:** 10.18632/oncotarget.24276

**Published:** 2018-01-19

**Authors:** Sinead A. Noonan, Maria E. Morrissey, Petra Martin, Monika Biniecka, Shane Ó'Meachair, Aoife Maguire, Miriam Tosetto, Blathnaid Nolan, John Hyland, Kieran Sheahan, Diarmuid O'Donoghue, Hugh Mulcahy, David Fennelly, Jacintha O'Sullivan

**Affiliations:** ^1^ Centre for Colorectal Disease, St. Vincent's University Hospital, University College Dublin, Dublin, Ireland; ^2^ Trinity Translational Medicine Institute (TTMI), Department of Surgery, Trinity College Dublin, St James's Hospital, Dublin, Ireland; ^3^ Education and Research Centre, St. Vincent's University Hospital, University College Dublin, Dublin, Ireland; ^4^ Centre for Health Decision Science (CHeDS), School of Computer Science and Statistics, Trinity College Dublin, Dublin, Ireland; ^5^ Department of Histopathology, St. James's Hospital, Dublin, Ireland

**Keywords:** bevacizumab, predictive biomarkers, metastatic colorectal cancer, vasculature, inflammation

## Abstract

Despite treatment of patients with metastatic colorectal cancer (mCRC) with bevacizumab plus chemotherapy, response rates are modest and there are no biomarkers available that will predict response. The aim of this study was to assess if markers associated with three interconnected cancer-associated biological processes, specifically angiogenesis, inflammation and oxidative damage, could stratify the survival outcome of this cohort.

Levels of angiogenesis, inflammation and oxidative damage markers were assessed in pre-bevacizumab resected tumour and serum samples of mCRC patients by dual immunofluorescence, immunohistochemistry and ELISA.

This study identified that specific markers of angiogenesis, inflammation and oxidative damage stratify survival of patients on this anti-angiogenic treatment. Biomarkers of immature tumour vasculature (% IMM, p=0.026, n=80), high levels of oxidative damage in the tumour epithelium (intensity of 8-oxo-dG in nuclear and cytoplasmic compartments, p=0.042 and 0.038 respectively, n=75) and lower systemic pro-inflammatory cytokines (IL6 and IL8, p=0.053 and 0.049 respectively, n=61) significantly stratify with median overall survival (OS).

In summary, screening for a panel of biomarkers for high levels of immature tumour vasculature, high levels of oxidative DNA damage and low levels of systemic pro-inflammatory cytokines may be beneficial in predicting enhanced survival outcome following bevacizumab treatment for mCRC.

## INTRODUCTION

Colorectal cancer (CRC) is the third most frequent cause of cancer death worldwide, responsible for over six hundred thousand deaths per year [1]. About 25% to 30% of CRC patients present with metastases at diagnosis [2]. Despite the introduction of molecularly targeted therapies, the five year survival rate of metastatic CRC remains only 12% [3]. Targeted therapies include cetuximab (Erbitux) which targets the epithelial growth factor (EGF) receptor, and bevacizumab (Avastin) which is a humanized monoclonal antibody that targets the vascular endothelial growth factor (VEGF) to prevent it binding to its receptors [4]. Treatment with targeted therapies has improved progression free survival (PFS) in the case of cetuximab and overall survival (OS) in the case of bevacizumab [2]. However less than 50% of patients respond to these drugs and although KRAS mutation status predicts response to cetuximab, there is only limited or inconclusive data on biomarkers for predicting response to bevacizumab [2, 5–8]. Bevacizumab plus chemotherapy is a standard of care treatment for mCRC, and many patients receive this expensive targeted therapy, which can cause significant side-effects including hypertension, proteinuria and bowel perforation, without achieving a response [9]. Thus discovery of biomarkers that can predict response to bevacizumab are warranted for the informed and cost-effective management of patients with mCRC.

VEGF plays multiple roles – it acts on endothelial cells and is involved in the stimulation of endothelial mitogenesis, promotion of endothelial cell survival, control of vascular permeability, stimulation of the expression of factors that are involved in the degradation of the extra-cellular matrix, endothelial cell migration and the synthesis of erythropoietin [10, 11]. In addition, VEGF in the tumour microenvironment has been linked to the inhibition of dendritic cell maturation. Levels of dendritic cell inhibition induced by the microenvironment correlate with survival of CRC patients following bevacizumab treatment [12, 13]. VEGF and microvascular density (MVD) have been implicated as prognostic biomarkers for CRC patients [14]. Despite the fact that VEGF is the dominant factor in angiogenesis and its targeting has been an effective therapeutic strategy in CRC, pre-treatment levels of circulating VEGF levels in phase III clinical trials do not predict response to bevacizumab, nor are tumour expression levels of VEGF protein predictive of benefit to bevacizumab therapy in mCRC patients [15, 16]. Genetic polymorphisms in VEGF signalling factors have been proposed as potential predictive markers for bevacizumab [5, 17].

Inflammation and angiogenesis are two closely associated processes capable of potentiating each other. Angiogenic factors have dual functionality with both pro-angiogenic and pro-inflammatory functions while pro-inflammatory cytokines drive inflammation that potentiates angiogenesis thus favouring neoplastic growth [18]. CRC is an inflammation-driven malignancy. The important role of inflammation in carcinogenesis has been increasingly recognised with proliferation and metastasis paralleling normal biological processes of the immune system [18]. Inflammation leads to cytokine production, chemotaxis, increased vascular permeability, angiogenesis, cell proliferation, cell growth and cell mobilisation [19]. Chronic inflammation is also associated with increased oxidative stress and induction of oxidative stress in turn induces pro-inflammatory effects [20]. Oxidative stress leading to DNA damage has long been implicated in carcinogenesis and can drive cancer progression leading to a cycle of on-going damage [21, 22]. It is considered an initiating factor in driving angiogenesis in an inflammatory environment and we have shown that levels of oxidative damage correlate with survival of CRC patients [23–25].

The overall aim of this study was to examine if panels of markers associated with angiogenesis, oxidative stress and inflammation could stratify survival rates of mCRC patients subsequently treated with bevacizumab. We investigated the levels of markers associated with tumour vasculature, oxidative stress and inflammation in resected tumour tissue and serum samples taken prior to commencement of bevacizumab plus chemotherapy to establish their association with survival in metastatic patients following bevacizumab treatment.

## RESULTS

The aim of this study was to determine if specific markers of angiogenesis, inflammation and oxidative stress, stratified survival of CRC patients treated with bevacizumab plus chemotherapy. One or more markers of each of these processes were found to significantly stratify survival by univariate and multivariate analysis in either tumour tissue or in serum (Table 1–2, Supplementary Table 1).

**Table 1:** Outputs of statistical analyses for tissue scoring for vasculature, oxidative damage, infiltrating immune cells, and proliferation highlight vasculature immaturity (% IMM) and specific 8-oxo-dG staining patterns stratify overall survival rates

Biological Process	Tissue Marker	n	Above Median	Raw Univariate p-value	Adjusted p-value	Lasso Coefficient
**Vasculature**	Tumour MVD	80	0.150	0.176	0.609	−0.100
	**Tumour %IMM**		**0.026**	**0.006**	0.175	**-0.245**
	Normal MVD		0.725	0.956	0.997	-
	Normal % IMM	79	0.948	0.656	0.997	-
**Oxidative Damage**	8-oxo-dG EN%	75	0.526	0.701	0.997	-
	8-oxo-dG EC%		0.709	0.324	0.871	-
	8-oxo-dG SN%		0.434	0.948	0.997	-
	8-oxo-dG SC%		0.010	0.030	0.356	-
	**8-oxo-dG ENI**		**0.042**	**0.040**	0.385	**-0.574**
	**8-oxo-dG ECI**		**0.038**	**0.014**	0.284	**-0.056**
	8-oxo-dG SNI		0.543	0.541	0.983	-
	8-oxo-dG SCI		0.822	0.025	0.356	−0.736
	4HNE EN%		0.458	0.210	0.616	-
	4HNE EC%		0.303	0.098	0.487	-
	4HNE SN%		0.870	0.509	0.954	-
	4HNE SC%		0.300	0.193	0.609	-
	4HNE ENI		0.543	0.409	0.928	-
	4HNE ECI		0.255	0.103	0.487	−0.541
	4HNE SNI		0.450	0.625	0.997	-
	4HNE SCI		0.761	0.114	0.487	-
**Immune cells**	CD3	79	0.741	0.450	0.931	-
	CD68	78	0.812	0.778	0.997	-
**Proliferation**	Ki67 EN%	75	0.558	0.887	0.997	-
	Ki67 SN%		0.964	0.334	0.871	-

The statistical output columns show p-values from a univariate analysis when data is dichotomised into above and below median levels for survival curves, p-values from Cox proportional hazards for testing the association between the actual marker versus changes in survival with both raw univariate p-values and adjusted p-values from a multiple penalised regression analysis and non-zero lasso coefficients from multivariate analysis. p values ≤0.05 were considered significant and the numbers of patients scored are indicated. Markers found to be significant for survival curves were also significant by at least one additional statistical test, with 8-oxo-dG SC% also significant by a raw univariate test, and with % IMM, 8-oxo-dG ENI and ECI also significant by a raw univariate test and multivariate test (highlighted in bold). Vasculature structure was examined for microvascular density (MVD) and percentage vasculature immaturity (% IMM). Oxidative damage markers examined were 8-Oxo-2'-deoxyguanosine (8-oxo-dG) and 4-hydroxy-2-nonenal (4HNE). For immune cell infiltrates, the markers included were CD3 as a T cell marker and CD68 as a monocytes/macrophage marker. Ki67 was included as a proliferation marker. Normal, normal adjacent tissue; EN%, epithelial nuclear percent positivity; EC%, epithelial cytoplasmic percent positivity; SN%, stromal nuclear percent positivity; SC%, stromal cytoplasmic percent positivity; ENI, epithelial nuclear intensity; ECI, epithelial cytoplasmic intensity; SNI, stromal nuclear intensity; SCI, stromal cytoplasmic intensity.

### Levels of tumour vasculature immaturity stratify survival of mCRC patients receiving bevacizumab treatment

**Figure 1:**
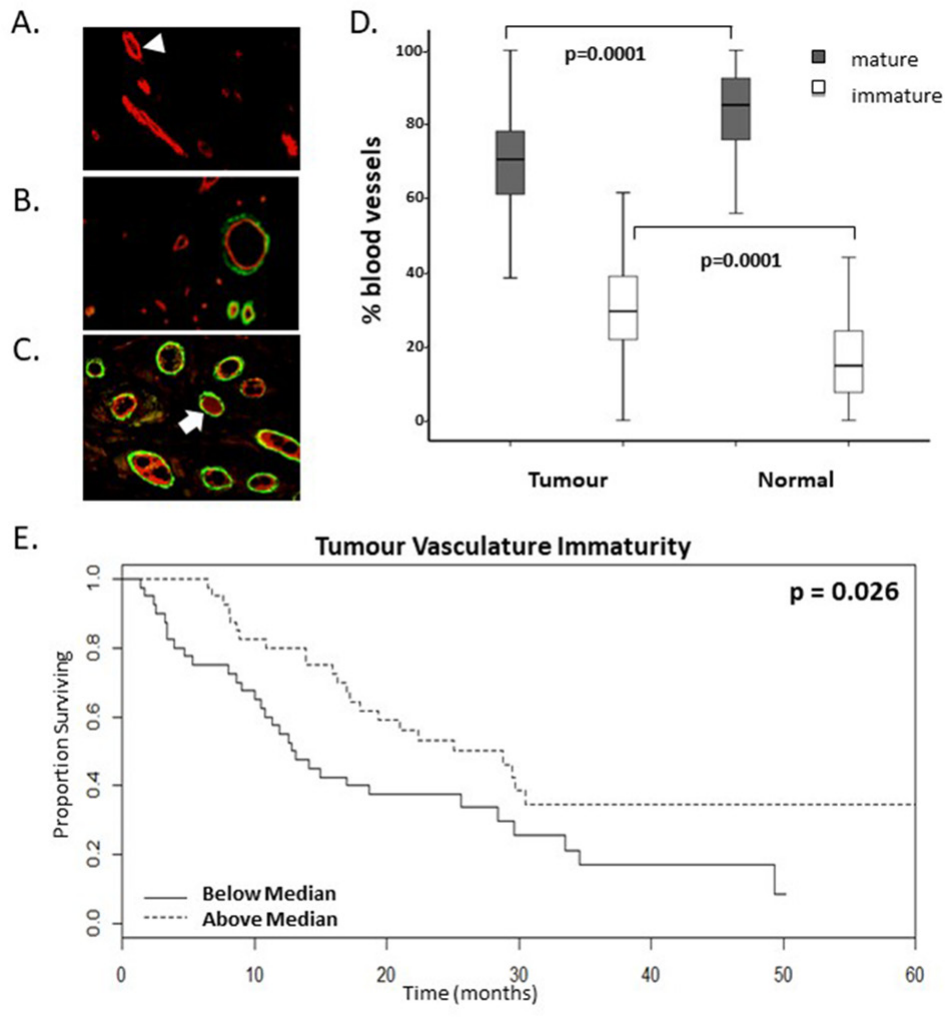
A higher proportion of immature vasculature correlates with enhanced survival rates of mCRC patients following bevacizumab treatment **(A-C).** Representative images of dual immunofluorescent staining for FVIII (red) and α-smooth muscle actin (αSMA) (green) indicative of pericyte recruitment to mature vessels. Varying levels of vasculature maturity was observed from (A.) low, (B.) moderate and (C.) high levels of vasculature maturity in the resected colorectal cancer tumour microenvironment. Arrowhead highlights an immature vessel; arrow highlights a mature vessel which has recruited pericytes. **D.** Graph shows the proportion of mature and immature vasculature in tumour tissue compared to matched normal tissue. There is a lower percentage of mature vasculature and a higher percentage of immature vasculature in tumour tissue compared to normal tissue (p values=0.0001, n=80). **E.** Kaplan-Meier survival curves of above (dotted line) and below (continuous line) median levels of percentage immature vasculature (% IMM) prior to commencing bevacizumab treatment versus overall survival in months (p value=0.026, n=80).

In order to distinguish mature and immature tumour vasculature in CRC tumours, levels of pericyte recruitment to vasculature in tumour resections prior to bevacizumab treatment were determined retrospectively. Figure 1A shows immature blood vessels in the tumour as indicated by FVIII staining (red) without any α-SMA staining (green). Figure 1B-1C show images of blood vessels that have recruited pericytes (co-stained red and green vessels). There is a higher proportion of mature and a lower proportion of immature blood vessels in tumour tissue compared to matched normal tissue (Figure 1D). Interestingly, our data show a significant stratification of overall survival (OS) based on the presence of higher levels of vasculature immaturity in the pre-bevacizumab treatment tumours (Figure 1E, Table 1, n=80, p=0.026 univariate test). When dichotomised according to the median levels of percentage immature vasculature, the surviving proportion of patients at 60-months or last observation with higher levels of % IMM was 0.32, whereas for patients with lower levels the surviving proportion was 0.09 (Figure 1E, Supplementary Table 2). In addition, this was significant both by Cox proportional hazards with a raw univariate p=0.006 and by multivariate analysis with a lasso coefficient of −0.245 (Table 1).

### Levels of oxidative damage in the tumour microenvironment stratify survival of mCRC patients receiving bevacizumab treatment

**Figure 2:**
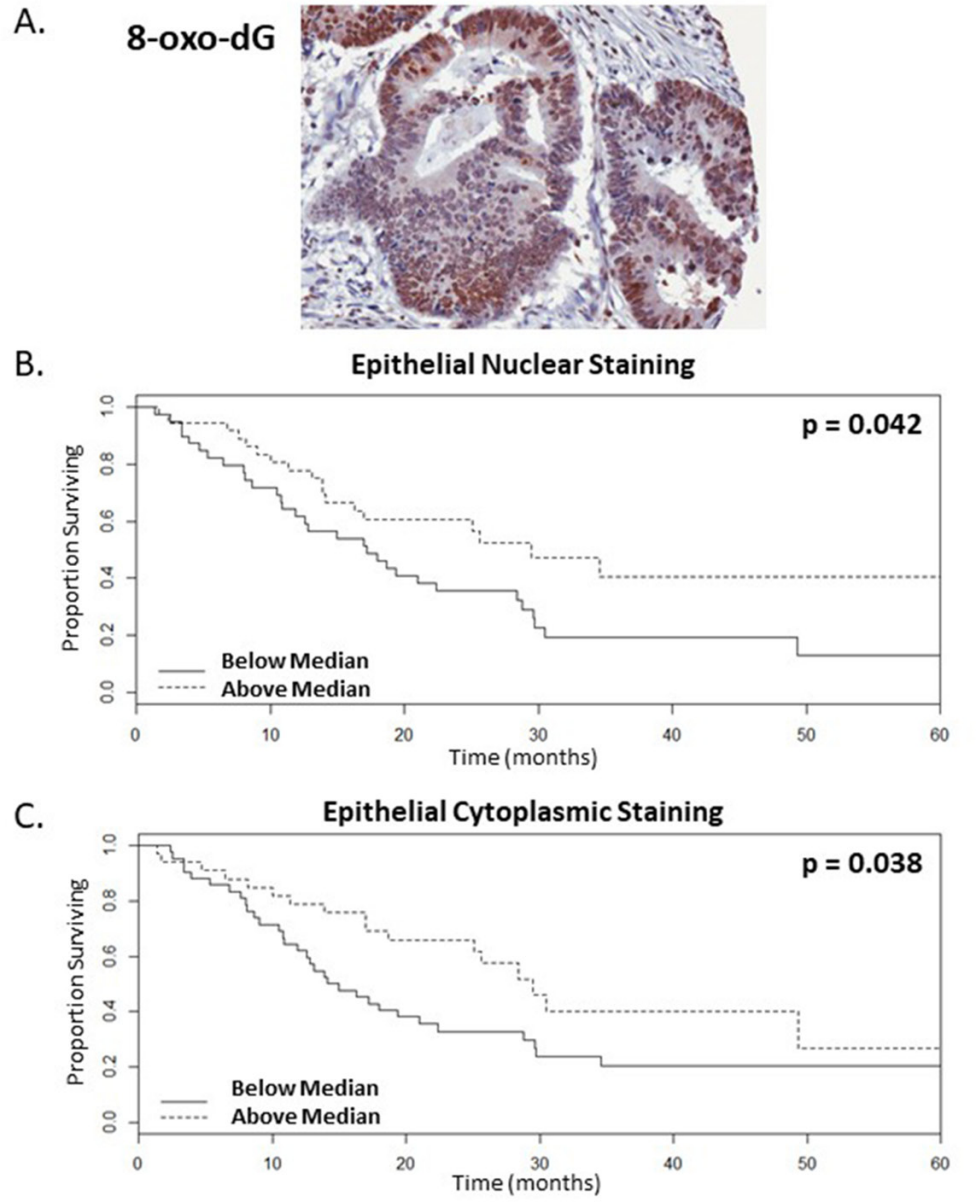
Higher levels of oxidative damage correlate with enhanced survival rates of mCRC patients following bevacizumab treatment **(A).** Representative image of resected colorectal tumour tissue showing strong epithelial and weak stromal staining for the oxidative damage marker, 8-oxo-dG **(B-C).** Kaplan-Meier survival curves of above (dotted line) and below (continuous line) median levels of epithelial staining intensity versus overall survival in months, where (B.) nuclear and (C.) cytoplasmic staining with p values=0.042 and 0.038 respectively, n=75.

As 8-oxo-dG is a predominant marker of oxidative stress and is linked to the presence of DNA adducts, the levels of this marker was assessed in the nucleus and cytoplasm of tumour tissue. There is a significant correlation between the presence of higher levels of oxidative damage in the tumour and patients that are likely to have enhanced OS following bevacizumab treatment. This was significant for oxidative damage in the epithelial compartment of the tumour, specifically epithelial nuclear intensity (ENI) and epithelial cytoplasmic intensity (ECI) (Figure 2, n=75, p=0.042 and 0.038 univariate test respectively). When dichotomised according to the median levels of oxidative damage staining, the surviving proportions of patients at 60-months or last observation with higher levels of ENI and ECI were 0.41 and 0.27 respectively, whereas for patients with lower levels the surviving proportions were 0.13 or 0.21 respectively (Figure 2B-2C, Supplementary Table 2). In addition, these were significant by Cox proportional hazards with a raw univariate p=0.040 and 0.014 respectively (Table 1), although these were not significant by a multiple correction adjusted p-value. 8-oxo-dG ENI and ECI were confirmed to be significant by multivariate analysis with lasso coefficients of −0.574 and −0.056 respectively.

### Serum levels of pro-inflammatory cytokines stratify survival of mCRC patients receiving bevacizumab treatment

**Figure 3:**
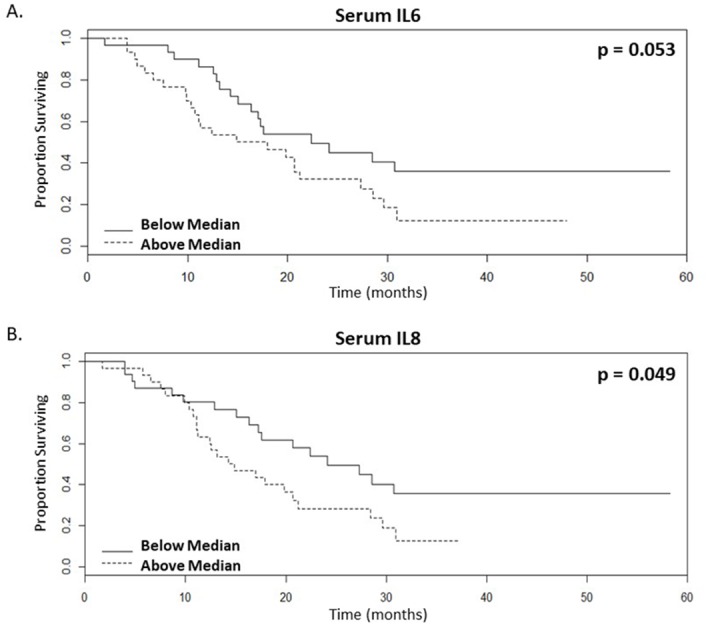
Lower serum levels of inflammatory cytokines correlate with enhanced survival rates of mCRC patients following bevacizumab treatment **(A-B).** Kaplan-Meier survival curves of above (dotted line) and below (continuous line) median levels of (A.) IL6 (p value=0.053) and (B.) IL8 (p value=0.049) in patient serum prior to commencing bevacizumab treatment versus overall survival in months, n=61.

Levels of pro-inflammatory cytokines were assessed in serum obtained prior to bevacizumab treatment. Our data shows a significant correlation between the presence of below median levels of IL6 and IL8 in pre-treatment serum and patients that are likely to have enhanced OS following bevacizumab treatment (Table 2, Figure 3, n=61, p=0.053 and 0.049 respectively, univariate test). When dichotomised according to the median levels of IL6 (Figure 3A) or IL8 (Figure 3B), the surviving proportions of patients at 60-months with lower levels of IL6 and IL8 were both 0.36, whereas for patients with higher levels the surviving proportions were 0.12 or 0.13 respectively (Figure 3, Supplementary Table 2). IL6 is also significant by Cox proportional hazards with a raw univariate p=0.003, a multiple-correction adjusted p=0.036 and by a multivariate analysis with a lasso coefficient of 0.205 (Table 2). Similarly, IL8 is also significant both by Cox proportional hazards with a raw univariate p=0.006, a multiple-correction adjusted p=0.036 and by a multivariate analysis with a lasso coefficient of 0.058 (Table 2). In addition, IL6 is significant by univariate and multivariate analyses with PFS as the endpoint (Supplementary Table 3).

**Table 2:** Outputs of statistical analyses for serum levels of angiogenic factors, oxidative damage markers and inflammatory cytokine levels indicate that IL6 and IL8 stratify overall survival rates

Biological Process	Serum Marker	n	Above Median	Raw Univariate p-value	Adjusted p-value	Lasso Coefficient
**Angiogenic Factors**	VEGF-A	61	0.748	0.144	0.296	-
	PDGFβ		0.481	0.159	0.296	-
	ANG2		0.138	0.091	0.296	-
	TGFβ1	57	0.913	0.653	0.849	-
**Oxidative Damage**	8-oxo-dG	60	0.483	0.489	0.794	-
	HEL		0.874	0.991	0.996	-
**Cytokines**	IL1β	61	0.529	0.116	0.296	-
	**IL6**		**0.053**	**0.003**	**0.036**	**0.205**
	**IL8**		**0.049**	**0.006**	**0.036**	**0.058**
	TNFα		0.748	0.094	0.296	-

The statistical output columns show p-values from a univariate analysis when data is dichotomised into above and below median levels for survival curves, p-values from Cox proportional hazards for testing the association between the actual marker versus changes in survival with both raw univariate p-values and adjusted p-values from a multiple penalised regression analysis and non-zero lasso coefficients from multivariate analysis respectively. p values ≤0.05 were considered significant and the numbers of patients scored are indicated. IL6 and IL8 were significant across all four statistical analyses (highlighted in bold).

Tissue biomarkers that did not stratify survival include MVD, tumour infiltrating T-cells and macrophages (CD3 and CD68 respectively), and the cell proliferation marker Ki67 (Table 1). Serum biomarkers that did not stratify survival were angiogenic factors (VEGFA, PDGFβ, ANG2 and TGFβ1), oxidative damage markers (8-oxo-dG and HEL) and several cytokines (IL1β and TNFα) (Table 2).

## DISCUSSION

This study provides evidence that markers of tumour biology, specifically vasculature immaturity (% IMM) and tumour epithelial 8-oxo-dG in the tissue and serum IL6 and IL8 stratify survival outcome of mCRC patients on bevacizumab treatment. Of our broad list of potential biomarkers investigated in this study, specific components of cancer-associated biological processes were identified as predictive biomarkers by univariate and multivariate analysis.

We found significant stratification for levels of vasculature maturity and OS following bevacizumab treatment. Our data shows that the presence of above median levels of immature vasculature in the tumour microenvironment is associated with enhanced survival. Pericytes are support cells that are closely apposed to the outer surfaces of the endothelial tubes, where they provide mechanical and physiological support to endothelial cells [19]. Acquisition of a pericyte layer in mature vasculature is essential for functional vascular patterning, diameter regulation and vessel stabilization [26]. VEGF is required to sustain immature vessels (vessels that have not acquired a pericyte layer) and so these vessels are susceptible to VEGF loss [27, 28]. This correlation with survival is specific to vasculature maturity and does not extend to overall levels of vascularisation in terms of MVD, in agreement with previous publications [29–31]. For expression of genes involved in vascular morphogenesis and maturation, it was reported that low VEGFB and high FLT1 in primary tumours and high ACVRL1 levels in liver metastases were associated with enhanced OS of CRC patients with liver metastases treated with bevacizumab plus chemotherapy [32]. Molecular approaches have identified some candidate biomarkers related to pericytes; high levels of tumour expression of a gene involved in pericyte-driven tumour maturation, EPHB4, was found to be associated with decreased OS of CRC patients treated with bevacizumab and pericyte germline polymorphisms in mCRC patients treated with bevacizumab plus chemotherapy are associated with clinical outcome [33, 34]. We hypothesise that in the tumour microenvironment of patients who are likely to have a poorer survival, this highly mature vasculature system may have progressed beyond a point whereby subsequent treatment with bevacizumab will be sufficient to bring about a meaningful clinical response. Whereas in patients who achieve a good response, predominantly early neovasculature or actively altering vessels are present and so these tumours may not have the mechanisms or structures in place to be resistant to bevacizumab.

We found significant stratification for levels of oxidative damage and OS following bevacizumab treatment. Our data show that the presence of above median levels of oxidative damage in tumours is associated with enhanced survival. 8-oxo-dG is one of the predominant forms of free radical-induced oxidative lesions in nuclear and mitochondrial DNA, and has been widely used as a biomarker for oxidative stress in cancer [35]. Inefficient tumour vasculature can cause significant oxidative stress through the production of reactive oxygen species [36, 37]. Studies of immature blood vessels in disease states such as inflammatory arthritis have indicated a relationship between blood vessel stability, hypoxia, oxidative DNA damage and persistent inflammation [23, 24]. High levels of reactive oxygen and nitrogen species produce 8-oxo-dG and have been observed in chronic inflammatory conditions such as ulcerative colitis, a condition which predisposes to CRC [38, 39]. A higher level of serum 8-oxo-dG is a risk factor for early colorectal cancer [40]. The occurrence of high levels of genomic damage has been reported in early tumorigenesis, including in colonic tissue, which is in line with the mutator phenotype hypothesis whereby high levels of genomic instability in cancer cells, including by oxidative damage, produces a pool of mutations, some of which confer a selective advantage [41]. It is reasoned that this may then be followed by selection away from prolonged severe genomic instability in more progressed tumours towards more moderate levels, due to likely cell lethality if this severe damage was to continue [41]. Thus it is possible that in our patient cohort with below median levels of this DNA damage indicator, the tumours may have more moderate levels of genomic instability possibly brought about by selective advantage, and/or the presence of a mature vasculature making these tumours more capable of evading the effects of oxidative stress. Oxidative stress produces both DNA damage, such as the formation of DNA adducts, e.g. 8-oxo-dG, and lipid damage, such as 4-HNE which is an inducer and mediator of oxidative stress. Our findings show that while specific staining patterns of 8-oxo-dG in the tumour microenvironment significantly correlated with survival, 4-HNE did not stratify patient survival, possibly indicating that DNA adduct formation may be more important than lipid peroxidation in contributing to treatment outcome.

We found significant stratification for levels of pro-inflammatory cytokines and OS following bevacizumab treatment. Our data shows that the presence of below median levels of IL6 and IL8 in pre-bevacizumab treatment serum is associated with enhanced survival. Systemic inflammation markers are typically elevated in cancer patients and other diseases. IL6 is a proinflammatory cytokine with a typical protumorigenic effect, and serum levels are elevated in cancer patients [39]. Functionally, IL6 is involved in inflammatory and costimulatory action, inducing proliferation, differentiation and synergizing with TGFβ to drive Th17. IL8 is a chemokine that attracts neutrophils, basophils and T-lymphocytes and is involved in neutrophil activation [42]. Circulating inflammatory cytokines in many cancers are associated with poor prognosis. In line with our findings, high pre-treatment levels of serum IL-6 have recently been associated with poorer OS and PFS in mCRC [43]. Elevated serum IL8 has been proposed as a biomarker for diagnosis, poor prognosis and disease progression in CRC [44]. There is some evidence of IL6 and IL8 as prognostic biomarkers of survival in CRC patients and as predictive markers for response to bevacizumab treatment in renal cell carcinoma [45–47]. Lower serum levels of IL6 and IL8 have been associated with a higher likelihood of response in clinical outcomes of mCRC patients treated with irinotecan plus bevacizumab [48]. IL6-stimulated vessel sprouts have defective pericyte coverage compared with VEGF-stimulated vessels in an *ex vivo* animal aortic ring assay [49]. Thus IL6 stimulation has been linked with defective pericyte coverage and our findings show that both lower levels of pericyte coverage in the tumour vasculature and lower systemic IL6 levels are biomarkers for predicting enhanced survival following treatment with bevacizumab. It is possible that treatment with bevacizumab may be insufficient to counter the likely pleotropic roles of these inflammatory pro-tumorigenic cytokines. Markers were also investigated in terms of stratification of Progression-Free Survival (PFS), however only IL6 of the serum markers and none of the tissue markers were significant by both univariate and multivariate analyses (Supplementary Table 3 for serum markers, data not shown for tissue markers).

Factors suggested to be associated with a response to bevacizumab-based therapy in CRC patients include longer PFS, low LDH levels, KRAS wild-type status, good performance status (PS) and a single site of metastasis [8]. While many of the potential biomarkers we tested are known or are likely to be important features of CRC tumours, there have been limited findings in terms of stratification of patient OS. For example, while VEGF is certainly a crucial factor in angiogenesis and several studies have indicated circulating VEGF as a prognostic biomarker in CRC, the lack of predictive value of proangiogenic proteins in our study is consistent with prior studies that failed to demonstrate the predictive ability of biomarkers including VEGF for anti-VEGF therapy in CRC patients [7, 15, 45, 47]. Large interindividual variations in angiogenic biomarkers and redundancy within family members may be a reason for the difficulty in the identification of angiogenic pathway-based predictive biomarkers [50]. Although contradictory data exists, high expression of VEGF D in tumour tissue has been indicated as predictive of resistance to bevacizumab-based therapy of mCRC patients in terms of PFS [51]. Combining VEGFA, FLT1, and KDR expressions has been shown to be associated with a poor CRC prognosis and poor response to bevacizumab treatment [52]. Soluble VEGFR-1 plasma levels have been indicated as predictive for survival of unresectable advanced CRC patients treated with bevacizumab plus chemotherapy [53]. Based on proteomics screening, we showed that levels of three proteins with roles in regulating angiogenesis – apolipoprotein E, vitamin D binding protein and angiotensinogen, are associated with survival outcomes of mCRC patients treated bevacizumab plus chemotherapy [54]. Various tumour biomarkers that associate with outcome of CRC patients treated with a bevacizumab-based therapy have been reported, such as NOTCH1 which associates with poorer survival [55]. Other types of putative biomarkers have been reported that associate with outcome, such as genetic polymorphisms including in FLT1, levels of a number of microRNAs, systemic vasoactive peptides and circulating endothelial cells [56–66]. Despite no correlation found in this study with survival and CD3 or CD68 immune cells, other characteristics of the immune system may warrant investigation as predictive biomarkers. Indeed levels of dendritic cell inhibition by the tumour microenvironment following bevacizumab treatment have been found to correlate with survival of mCRC patients [13]. Also, variations in some genes regulating tumour-associated macrophage-related functions may predict outcomes of bevacizumab treatment [67].

**Figure 4:**
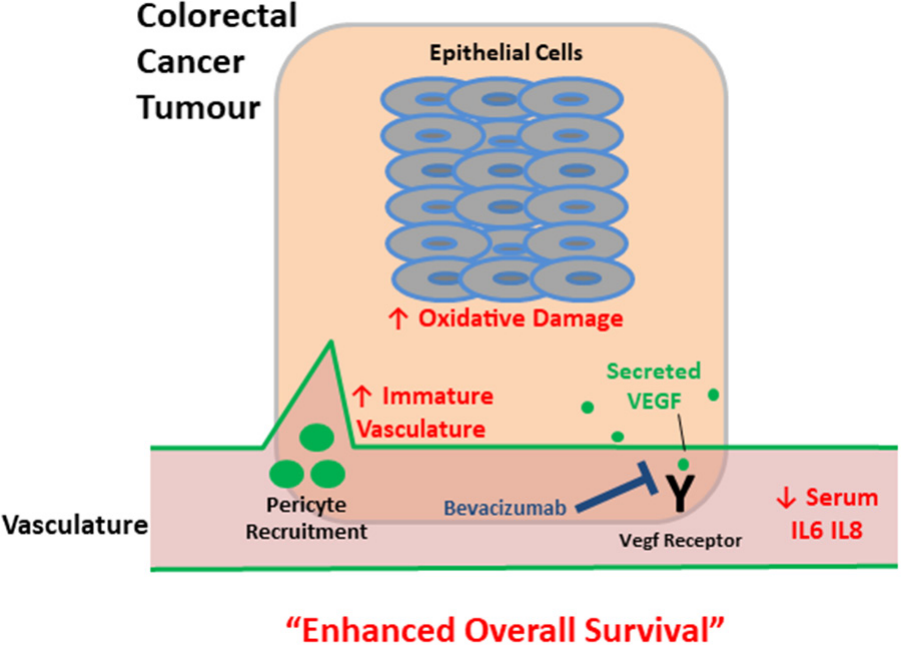
Schematic depicting tumour and serum biomarkers that stratify survival in metastatic colorectal cancer patients following treatment with bevacizumab Up and down arrows indicate that altered levels (higher and lower respectively) of these biomarkers (red text) correlate with enhanced overall survival – a higher proportion of immature tumour vasculature, increased oxidative damage and lower levels of circulating inflammatory cytokines. For patients that have a lower survival, it is possible that these interconnected cancer-associated biological processes may have progressed beyond a point whereby subsequent treatment following surgery with anti-VEGF therapy is sufficient to enhance patient survival levels.

Accordingly we propose a colorectal cancer model predictive of mCRC patient survival following bevacizumab treatment (Figure 4). We have constructed a model predictive of enhanced overall survival of mCRC patients on this anti-angiogenic treatment as stratified by biomarkers for (i) higher levels of immature tumour vasculature, (ii) higher levels of tumour epithelial oxidative damage and (iii) lower levels serum inflammatory cytokine (Figure 4). Angiogenesis, inflammation and genome instability are hallmarks of cancer and there is a well-established epidemiological link between CRC and inflammatory disease. For patients that have a lower survival, it is possible that these cancer-associated biological processes may have progressed beyond a point whereby subsequent treatment following surgery with anti-VEGF therapy is sufficient to enhance patient survival levels, likely by promoting treatment resistance mechanisms. The local tumour environment of patients with a lower response to bevacizumab may produce high levels of other factors that effectively compensate for the lack of available VEGF, thus mediating angiogenesis and oxidative damage evasion, alongside circulatory inflammation further promoting mCRC. For example, we have previously shown that the local tumour microenvironment of mCRC patients contains high levels of the chemokines CXCL1, CXCL5, and CCL2 which have proangiogenic properties [68]. Thus, reduced survival on bevacizumab treatment may occur because of the failure of this therapy to directly or indirectly attenuate the effects of an already established mature vasculature, oxidative damage resistance mechanisms and systemic inflammation.

Confounding our findings on biomarkers predicting survival would be inconsistencies in chemotherapy backbone and later line treatments that our patient cohorts would have received. Therefore these statistically significant findings are hypothesis-generating for corroboration of these findings in controlled validation studies across multiple sites to confirm reproducibility and reliability of these predictive biomarkers in the clinic. It would also be of interest to determine if these biomarkers stratify survival of patients receiving other antiangiogenic treatments. In summary, we show that patients who are likely to have enhanced OS following bevacizumab treatment have high levels of immature vasculature and oxidative damage in the pre-treatment tumour microenvironment and lower systemic inflammatory cytokines, thus providing evidence that these represent predictive biomarkers of survival for patients receiving bevacizumab plus chemotherapy.

## MATERIALS AND METHODS

### Patient samples

Ethical approval to conduct all aspects of this work was granted by the Ethics Committee of St. Vincent's University Hospital. Formalin-fixed paraffin-embedded (FFPE) archival resected tumour tissue samples were accessed for 80 patients and serum samples for 61 patients with mCRC who were subsequently treated with bevacizumab plus chemotherapy between 2004 and 2009. Supplementary Table 1 shows the clinical details of this patient cohort. OS was calculated from the date of first treatment to the date of death or last follow up and PFS was calculated from date of diagnosis of metastatic disease until radiological progression.

### Immunofluorescence and immunohistochemical staining

Three tumour blocks and one matched normal block were obtained per patient. The tumour margin block was used as the normal control. Two tumour gross sections and one normal gross section were created per patient. Slides were deparaffinised, rehydrated and heated-antigen retrieval was performed in 1M citric acid. For dual immunofluorescence, sections were incubated with primary antibodies for rabbit anti-factor VIII (FVIII) (1:100) and mouse anti– α-smooth muscle actin (αSMA) (1:100) (Dako, Glostrup, Denmark), Cy2/Cy3-conjugated secondary antibody respectively (Jackson Immuno-Research, Suffolk, UK) and counterstained with DAPI nuclear stain (1:1000; Sigma, St. Louis, MO). Sections were mounted with anti-fade (Molecular Probes, Eugene, OR) and assessed by immunofluorescence microscopy. Images were processed using LSM imaging software (Zeiss, Oberkochen, Germany). Tissue microarrays (TMA) with a core size of 0.6mm were constructed from the FFPE tumour resections, with all tissue reviewed by a pathologist (A.M.) pre and post TMA construction. For immunohistochemistry, sections were incubated with primary antibodies for anti-8-oxo-dG (Genox, 1:40), 4HNE (Gentaur, 1:40) and Ki67 (Dako, 1:100), CD3 or CD68 (Dako, 1:25 and 1:1000 respectively), and secondary antibody horseradish peroxidise. Endogenous peroxidase activity was blocked using 3% hydrogen peroxide. Diaminobenzidine was used to visualize staining and sections were counter-stained with haematoxylin, dehydrated and mounted.

### Tissue scoring

For blood vessel maturity, five high power fields were scored per tissue block using two gross tumour sections and one normal adjacent gross section per patient equating to 10 fields scored for the tumour tissue and 5 fields scored for the matched normal tissue. Percentage blood vessel maturity was calculated as the number of blood vessels per high power field co-expressing FVIII and αSMA as a measure of mature blood vessels, relative to those expressing only FVIII as a measure of all blood vessels and indicative of immature blood vessels. For 8-oxodG and 4HNE, epithelial and stromal cells were assessed for both nuclear and cytoplasmic staining using percent positivity and staining intensity as scoring parameters. For Ki67, epithelial and stromal cells were assessed for nuclear percent positivity. Positive cell count was assessed using 6 categories (0%, 10%, 25%, 50%, 75%, 90% and 100%). Intensity was assessed using a scale of 0, 1, 2 and 3 which correlated with negative, weak, medium and strong staining, respectively. The mean value of each parameter was calculated and correlated with clinical outcome of the patients. All scoring was performed blinded to the clinical data.

### Serum analysis

Stored prospective serum samples were accessed for 61 CRC patients. Following informed consent, samples were taken prior to treatment with bevacizumab and stored at −80°C. Enzyme-linked immunosorbent assays (ELISAs) were performed on serum samples as per manufacturers’ guidelines as appropriate. IL8, IL6, IL1β and TNFα were measured by a multiplex ELISA using the Human Pro-Inflammatory II 4-Plex Assay kit (MSD). VEGF-A, Ang2 were measured by standard sandwich ELISA using a DuoSet ELISA kit (R&D Systems) and PDGFβ (PDGF-BB isoform) was measured using an ELISA development kit (PeproTech). TGFβ1 was measured using an ELISA Ready-SET-Go kit (eBioscience) on at least 5-fold diluted and acid-treated serum samples to activate TGFβ1. 4HNE and 8-oxo-dG were measured by competitive ELISAs (Genox).

### Statistical analysis

Apart from Wilcoxon matched-pairs signed-rank test as performed using GraphPad Prism for % IMM, all analysis was conducted using R software. Univariate tests of predictive factors were conducted using a log rank test for testing stratification by above or below median for survival curves and a Wald test of Cox proportional hazards to test the association between the actual marker values with changes in survival. Due to the large number of simultaneous tests, adjusted p-values corrected for multiple comparisons were included. A False Discovery Rate of 5% was applied to the univariate p-values. Penalised Cox proportional hazards were used for multivariate analysis. An L1 penalty was applied to coefficients corresponding to lasso Cox regression whereby coefficients are shrunken towards zero in order to give better predictive power and to remove noisy variables that are weakly predictive and correlated with other variables, as well as providing more stable variable selection than stepwise methods. This approach is standard practice where there are many markers relative to observed patients [69]. Markers were considered of interest when they were significant by at least one univariate analysis including when dichotomised by median for Kaplan-Meier survival curves, in addition to showing significance by multivariate analysis.

## CONCLUSION

This study to our knowledge identifies for the first time that biomarkers of immature tumour vasculature, high levels of oxidative DNA damage and lower systemic pro-inflammatory cytokines may be beneficial in predicting enhanced survival outcome following bevacizumab treatment.

## SUPPLEMENTARY MATERIALS TABLES



## Abbreviations

mCRC: Metastatic colorectal cancer
VEGF: vascular endothelial growth factor
FFPE: formalin-fixed, paraffin-embedded
TMA: tissue microarrays
OS: overall survival
