# Supplementary material for: Tumour vasculature immaturity, oxidative damage and systemic inflammation stratify survival of colorectal cancer patients on bevacizumab treatment
Source: Oncotarget. 2018 Jan 19;9(12):10536–48. doi: 10.18632/oncotarget.24276 (PMC5828217; doi:10.18632/oncotarget.24276)
Supplement: Supplementary file 1 [file oncotarget-09-10536-s001.pdf]

Tumour vasculature immaturity, oxidative damage and systemic inflammation stratify survival of colorectal cancer patients on bevacizumab treatment

SUPPLEMENTARY MATERIALS

Supplementaty Table 1: Patient demographics

Patient Demographics		Tissue Patients (n=80)		Serum Patients (n=61)	
Age (years)		64	(25-84)	62	(29-81)
Overall Survival (months)		38	(6-140)	33	(4-61)
PFS (months)		15	(1-52)	14	(1-59)
Gender	Male/Female	45/35	6:4	38/23	6:4
Dukes' Classification at Diagnosis	A	1	1%	2	3%
	B	13	16%	9	15%
	C	29	36%	15	25%
	D	37	46%	35	57%
Bevacizumab	First Line	65	81%	53	87%
	Maintenance	31	39%	23	38%
	Duration (months)	10.8	(0.4-38.4)	10.1	(0.4-41.5)
Liver Resection		19	24%	12	20%
Complete Remission		10	13%	9	15%

Patient demographics for tissue (n=80) and serum (n=61) scoring. These are not totally independent cohorts with 22 patients providing both tissue and serum samples of 119 patients in total. Within columns the value to the left indicates the number of patients and to the right is a range, ratio or percentage as appropriate.

Supplementaty Table 2: Probability of survival

Marker	Above Median	Below Median
% IMM	0.35	0.09
8-oxo-dG ENI	0.41	0.13
8-oxo-dG ECI	0.27	0.21
IL6	0.12	0.36
IL8	0.13	0.36

Probability of survival at 60 months or last observation.

Supplementaty Table 3: Outputs of statistical analyses for serum levels of angiogenic factors, oxidative damage markers and inflammatory cytokine levels indicate that IL6 stratifies Progression Free Survival (PFS) rates

Biological Process	Serum Marker	n	Above Median	Raw Univariate p-value	Adjusted p-value	Lasso Coefficient
**Angiogenic Factors**	VEGF-A	61	0.34	0.15	0.26	-
	PDGFβ		0.89	0.40	0.57	-
	ANG2		0.57	0.59	0.66	-
	TGFβ1	57	0.87	0.53	0.66	-
**Oxidative Damage**	8-oxo-dG	60	0.53	0.16	0.26	-0.09
	HEL		0.65	0.86	0.86	-
**Cytokines**	IL1β	61	0.43	0.07	0.17	-
	**IL6**		**0.02**	**0.03**	0.17	**0.01**
	IL8		0.01	0.06	0.17	-
	TNFα		0.51	0.04	0.27	-

The statistical output columns show p-values from a univariate analysis when data is dichotomised into above and below median, p-values from Cox proportional hazards for testing the association between the actual marker versus with changes in survival with both raw univariate p-values and adjusted p-values from a multiple penalised regression analysis and non-zero lasso coefficients from multivariate analysis respectively. p values ≤0.05 were considered significant and the numbers of patients scored are indicated. IL6 was significant for survival curves, by a raw univariate test and a multivariate test (highlighted in bold).
